# Disaster day: a simulation-based competition for educating emergency medicine residents and medical students on disaster medicine

**DOI:** 10.1186/s12245-023-00520-1

**Published:** 2023-09-13

**Authors:** Shayne Gue, Stephanie Cohen, Maria Tassone, Ayanna Walker, Andy Little, Martin Morales-Cruz, Casey McGillicuddy, David Lebowitz, Robert Pell, Ariel Vera, Steven Nazario, Darielys Mejias, Andrew Bobbett, Drake Dixon, Anines Quinones, Latha Ganti

**Affiliations:** 1 University of Central Florida College of Medicine, Orlando, FL USA; 2 Emergency Medicine Residency Program, UCF/HCA Florida Healthcare GME (Greater Orlando/Osceola), Kissimmee, FL USA; 3 Emergency Medicine Residency Program, AdventHealth Orlando, Orlando, FL USA

**Keywords:** Medical education, Gamification, Disaster medicine, Mass casualty incident, Simulation

## Abstract

**Background:**

Disaster medicine is a growing field within the specialty of emergency medicine, but educational training typically focuses on hospital drills or other educational strategies, such as didactics, simulation, or tabletop exercises. With the success of gamification in other medical education applications, we sought to investigate if a novel gamified curricular innovation would lead to improved test performance and confidence in the ability to manage a real mass casualty incident (MCI).

**Methods:**

This was a prospective observational study of medical students and emergency medicine residents who participated in a 4-h simulation-based competition consisting of 4 unique stations. Each station had learning objectives associated with the content taught. Learners completed a pre-event survey, followed by participation in the competitive gamification event, and subsequently completed a post-event survey. Differences between pre- and post-event responses were matched and analyzed using paired and unpaired *t* tests for medical knowledge assessments, the Mann–Whitney *U* test for perceptions of confidence in the ability to manage an MCI event, and descriptive statistics provided on perceptions of the effectiveness of this educational strategy.

**Results:**

We analyzed data from 49 learners with matched (and unmatched) pre- and post-event survey responses. There was a statistically significant increase in medical knowledge assessment scores in both unmatched group means and available matched data (47 to 69%, *p* < 0.01, and 50 to 69%, *p* < 0.05). Self-reported confidence in the ability to handle an MCI scenario also significantly increased (*p* < 0.01). Finally, 100% of respondents indicated they “agreed” or “strongly agreed” that the event was an effective education tool for disaster preparedness and training.

**Conclusions:**

In this study, we found that learners perceived a novel gamification event as an effective educational tool, which led to improved learner knowledge and self-reported confidence in the ability to manage a real MCI.

## Background

A mass casualty incident (MCI) is “an event that overwhelms the local healthcare system, where the number of casualties vastly exceeds the local resources and capabilities in a short period of time” [1]. MCIs are increasing in number and severity across the USA and the world [2–5]. In 2022 alone, there were nearly 400 disaster events worldwide resulting in more than 30,000 deaths [6]. MCIs include natural disasters, pandemics, transportation incidents, acts of terrorism, military actions, and chemical, biological, or radiological events [7].

With the increasing incidence of MCIs in recent decades, there has been a focused interest from the federal government in preparing local resources, including healthcare systems, to respond to disasters [7]. In 2002, the Department of Homeland Security developed specific guidelines for the utilization of disaster exercises or “drills” to assist in preparation for mass casualty events [8]. These drills focus on system-level preparedness and improvement in coordination among all stakeholders.

Emergency medicine physicians serve as the front line of the healthcare system and are increasingly involved in leadership and management in prehospital and emergency medical services (EMS), which includes disaster planning and management. The National Association of EMS Physicians currently reports 77 ACGME-accredited EMS fellowship programs in the USA [9]. Regardless of subspecialty training, emergency medicine physicians are integral to disaster response within and beyond the physical boundaries of the emergency department. Thus, emergency medicine residents should receive focused training to respond in disaster settings [10]. Prior research has indicated that dedicated training improves performance on knowledge exams and in simulation scenarios [11]. Further, healthcare professionals indicate that participating in simulation events and other MCI training exercises improves disaster preparedness on the system level and confidence in the ability to perform their roles on the individual level [12–15]. Despite this, there is limited data and wide variability in the approach to education for emergency medicine residents and medical students [16].

Currently, didactic lectures, simulation, and hospital-wide training drills are the main educational strategies employed [17–19]. Our team realized the opportunity to incorporate educational gamification into the disaster preparedness training. Gamification is simply the “use of game design elements in non-game contexts” and has been exponentially increasing in educational realms [20–29]. In graduate medical education, we have observed that gamification leads to improved motivation among resident learners [30]. While there have been small-scale (with regard to the amount of resources needed, not the number of participants) exercises incorporating components of gamification in disaster education, there have been no large-scale (higher resource) gamified events utilizing these elements to train emergency medicine residents and students [31, 32].

As the field of disaster medicine continues to grow within the specialty of emergency medicine, there are opportunities for innovation in the way healthcare professionals are trained to provide care in such events. Incorporating large-scale gamified events for MCI training has the potential to motivate learners by creating an interactive and enjoyable method for learning.

### Game design

Disaster Day 2022 was a collaborative simulation/gamification event involving two ACGME-accredited 3-year, suburban, community-based emergency medicine residency programs in Orlando, Florida. The purpose of this event was to build collaborative relationships, foster physician wellness and networking, and accomplish specific educational goals pertinent to the emergency medicine physician in training.

The event took place on September 15, 2022, aligning with the protected weekly conference time, meaning residents in both programs have mandatory conference attendance when on the majority of rotations within their respective curricula. The overarching educational goal was to familiarize residents and medical students with the processes for pre-hospital care and system coordination for disaster medicine. Learners participated in a variety of live and simulated scenarios involving Simple Triage and Rapid Treatment (START) triage protocol, disaster resource allocation and utilization, field command, trauma resuscitation in resource-limited environments, and online medical control. The theme of the day was “Music Festival,” and each station or scenario was tied to potential catastrophes that could result during the event.

Residents and medical students were divided into 6 equal teams with learners of all levels. Teams rotated through a series of stations, each with three specific educational objectives (see Table 1), and earned points for correct and timely completion of assigned tasks. Prizes were awarded to the winning teams at each of the stations. The stations included (1) *Disaster Trivia*, a knowledge recall competition between 3 teams simultaneously utilizing “jeopardy”-style quizzing; (2) *START Triage*, a simulation-based race where learners had to correctly apply the START triage algorithm to patients encountered in a large field; (3) *Disaster Sim*, a multi-patient simulation where identified team leaders were blindfolded and resources became limited; and (4) *MCI Escape Room*, a music-festival themed escape room focused on resource allocation, triage, and management of environmental disorders. Each station was facilitated by multiple faculty members from both programs.

**Table 1:** Objectives

Station 1: Disaster Trivia
By the end of this session, residents will be able to: 1. Develop a structured approach to answering board-style questions on emergency medical services and disaster/event medicine content 2. Collaborate and gain consensus among interprofessional teams 3. Demonstrate leadership and teamwork in an approach to the management of patients in MCI and resource-limited scenarios
Station 2: START Triage
By the end of this session, residents will be able to: 1. Define a mass casualty incident (MCI) and discuss the unique challenges inherent to mass casualty incidents and disaster/event medicine 2. Differentiate between day-to-day triage and triage during a mass casualty incident 3. Apply the components of START (Simple Triage and Rapid Treatment) for mass casualty incidents
Station 3: Disaster Sim
By the end of this session, residents will be able to: 1. Evaluate and triage multiple patients from a natural disaster while remaining calm and utilizing the resources and team members you have 2. Assign roles in order to care for all patients effectively 3. Communicate effectively due disability to the team leader so the team can continue to treat patients effectively
Station 4. MCI Escape Room
By the end of this session, residents will be able to: 1. Manage patients they may encounter in a real-life event medicine situation such as a music festival 2. Formulate a plan to efficiently use limited resources available at events 3. Demonstrate leadership and teamwork to effectively treat patients outside of the hospital

## Methods

This was a cross-sectional prospective study analyzing the performance of emergency medicine residents and medical students in the application of medical knowledge relating to mass casualty incidents before and after a gamification event. This event was a part of the regular conference curriculum for two local emergency medicine residency programs. Therefore, attendance was required for residents and rotating students at both programs.

Prior to the event, participants voluntarily completed a pre-event survey, which included eight multiple-choice questions (MCQs) to evaluate medical knowledge as well as survey items pertaining to confidence in their ability to handle a disaster situation. After the event, participants voluntarily completed a post-event survey, which included seven MCQs to test medical knowledge as well as survey items regarding confidence in their ability to handle a disaster situation as well as the effectiveness of the gamified event as an educational strategy. Both of these surveys were adopted from a study at Rutgers New Jersey Medical School which previously found no significant difference in medical knowledge before and after a table-top disaster exercise for emergency medicine residents [33].

The medical knowledge MCQs of both pre- and post-event surveys were analyzed separately from the survey items evaluating resident perceptions regarding confidence in their ability to handle a similar situation as well as the effectiveness of the educational strategy. Paired and unpaired *t* tests were utilized to determine differences in mean scores on medical knowledge-based questions before and after the event. The Mann–Whitney *U* test was applied to determine the statistical significance of data relating to learner confidence. Descriptive statistics are provided for the post-event survey question relating to learner perceptions regarding the effectiveness of this gamified event as an educational strategy.

## Results

A total of 49 learners participated in the event: 19 medical students (3rd and 4th year students included), 12 PGY1s, 10 PGY2s, and 8 PGY3s. There was a 100% response rate for the pre-event survey and a 59% (29 of 49) response rate for the post-event survey. Given the lower response rate on post-event survey, we utilized both paired and unpaired *t* tests to determine significance. Assessments were created in line with Kirkpatrick’s model, focusing on levels of reactions, learning, and behavior [34].

For medical knowledge questions, we first utilized an unpaired *t* test to compare group means for pre- and post-event surveys from the total group as well as each learner level. The overall unpaired group means increased significantly between pre- and post-event surveys (47 to 69%, *p* < 0.01). Similar increases were seen for each learner level, represented in Table 2 below. A paired *t* test was performed to compare means for the group of learners completing both pre- and post-event surveys, which also indicated a statistically significant improvement in medical knowledge (50 to 69%, *p* < 0.05). See Table 3 below.

**Table 2:** Unpaired *t* test for medical knowledge questions

Total Learners		Students		PGY-1s		PGY-2s		PGY-3s	
Pre	Post	Pre	Post	Pre	Post	Pre	Post	Pre	Post
*N* = 49	*N* = 29	*N* = 19	*N* = 10	*N* = 12	*N* = 9	*N* = 10	*N* = 7	*N* = 8	*N* = 3
*M* = 0.47	*M* = 0.69	*M* = 0.31	*M* = 0.60	*M* = 0.54	*M* = 0.68	*M* = 0.60	*M* = 0.80	*M* = 0.56	*M* = 0.81
SD = 0.22	SD = 0.20	SD = 0.15	SD = 0.19	SD = 0.23	SD = 0.19	SD = 0.20	SD = 0.18	SD = 0.13	SD = 0.22
	*p* < 0.01		*p* < 0.05		*p* = 0.07		*p* < 0.05		*p* < 0.05

**Table 3:** Paired *t* test for medical knowledge questions

Total	Pre	Post
*N* = 29	*M* = 0.50	*M* = 0.69
DF = 28	SD = 0.20	SD = 0.20
*p* < 0.05	Mean of difference = 0.19	
	SD of difference = 0.27	

For the learner perception questions, we utilized the Mann–Whitney *U* test to determine statistical significance. Learners were given a scenario of a train derailment with 70 potential victims. Of the 49 learners completing the pre-event survey, most indicated they were neutral (47%, *n* = 23) or disagreed (45%, *n* = 22) with the statement “I am confident in my ability to handle an incident such as this.” Of the 29 learners completing the post-event survey, the majority indicated they agreed (66%, *n* = 19) with the statement, while 8 were neutral and 2 indicated disagreement. The Mann–Whitney *U* test indicated a statistically significant increase in confidence in post-event analysis (*p* < 0.01). See Table 4 below.

**Table 4:** Mann–Whitney *U* test for learner confidence (unpaired)

Question: I am confident in my ability to handle an incident such as this				
Responses	Pre	Post		
5 = Strongly agree	0	3	Sum of Ranks	3081
4 = Agree	4	16	Mean of Ranks	39.5
3 = Neutral	23	8	SD	96.72
2 = Disagree	17	2	U	227
1 = Strongly disagree	5	0	Z	-4.99
			*p* < 0.01	
Question: The subject of disaster medicine is important to emergency medicine				
Responses	Pre	Post		
5 = Strongly agree	30	18	Sum of Ranks	3081
4 = Agree	10	11	Mean of Ranks	39.5
3 = Neutral	7	0	SD	96.72
2 = Disagree	1	0	U	615
1 = Strongly disagree	1	0	Z	-0.98
			*p* = 0.16	

Finally, the post-survey included an item to elicit learner perceptions regarding the effectiveness of this gamified event as an educational strategy for learning disaster medicine. One hundred percent of respondents indicated they “agree” or “strongly agree” with the statement “I believe this Disaster Day exercise is an effective education tool for disaster preparedness and training.” See Table 5 below.

**Table 5:** Descriptive statistics for effectiveness of educational strategy

Question: *I believe this Disaster Day exercise is an effective education tool for disaster preparedness and training*									
Total learners		Students		PGY-1s		PGY-2s		PGY-3s	
Mean	4.690	Mean	4.7	Mean	4.889	Mean	4.571	Mean	4.333
SE	0.087	SE	0.153	SE	0.111	SE	0.202	SE	0.333
Median	5	Median	5	Median	5	Median	5	Median	4
Mode	5	Mode	5	Mode	5	Mode	5	Mode	4
SD	0.471	SD	0.483	SD	0.333	SD	0.535	SD	0.577
Range	1	Range	1	Range	1	Range	1	Range	1
Min	4	Min	4	Min	4	Min	4	Min	4
Max	5	Max	5	Max	5	Max	5	Max	5
Sum	136	Sum	47	Sum	44	Sum	32	Sum	13
Count	29	Count	10	Count	9	Count	7	Count	3

## Discussion

Emergency medicine physicians serve on the front lines of the healthcare system and are increasingly involved in the leadership and administration of EMS and disaster response. Further, the American Board of Emergency Medicine (ABEM) recognizes this importance and identifies understanding and applying “the principles of disaster and mass casualty management including preparedness, triage, mitigation, response, and recovery” in the Model of Clinical Practice of Emergency Medicine [35]. Therefore, emergency medicine residents must be educated on these principles.

Despite the documented need for education in this domain, most residency curricula rely on either system-wide drills with little focus on specific educational objectives or traditional pedagogy with a lecture-based approach. Gamification has been increasingly utilized in undergraduate and graduate medical education, with emergency medicine leading the way [21–29].

Our team designed and implemented a novel, gamified curricular innovation to educate emergency medicine residents and medical students about the core tenets of disaster management. The event included multiple stations, with unique educational objectives, tasked to better prepare learners of all levels to provide care and leadership in a real MCI. Gamification motivates learners by increasing engagement, granting autonomy, and allowing learners to demonstrate competence, in line with self-determination theory [36]. As hypothesized, learners overwhelmingly agreed that it was an effective educational strategy and improved confidence in their ability to manage a true mass casualty scenario. More importantly, learners demonstrated improved application of medical knowledge through increased scores on multiple-choice questions.

Several limitations exist. First, this was a large-scale, competition-based event, including nearly 50 learners with more than a dozen faculty facilitators from two emergency medicine residency programs and an affiliated medical school**.** Generalizability could be further limited by the requirements for the faculty time to develop the curricula, the initial cost of materials, and the space to conduct a large-scale event with multiple stations, including high-fidelity simulation. Further, we only evaluated medical knowledge immediately after the event and not at some pre-determined future point. Previous research has indicated there is a potential for decay of knowledge and skills when not actively utilized or practiced [37]. This is a particular concern considering disaster medicine is rarely applied in day-to-day practice. Future research may evaluate the retention of knowledge and skills over a longer period. Regardless, this event can be repeated each curricular cycle to mitigate this decay. Lastly, there was a significant decrease in the number of participants completing the post-event survey. The authors believe this was due to time constraints, as many learners were required to quickly return to clinical shifts following the event. Nevertheless, there exists a potential for selection/attrition bias, where participants less confident in their ability to perform may have opted not to participate in the post-event survey. We did note similar percentages of each subgroup (student versus PGY1 versus PGY2, etc.) did not complete the post-survey, which may limit the impact of this potential bias. Nonetheless, future studies should have more stringent methods to ensure a higher percentage of participants complete the post-survey assessment.

## Conclusions

We created and implemented a novel gamification event, “Disaster Day,” to educate emergency medicine residents and medical students on the core tenets of disaster medicine. Our results supported the hypothesis that learners participating in a large-scale gamification event would report increased confidence in their ability to handle a mass casualty scenario, agreement with the effectiveness of the gamified curricula as an educational strategy, and improved performance on a medical knowledge assessment. As a result, we plan to create, introduce, and evaluate the potential benefits of other gamified curricula to improve the development and retention of knowledge and skills across other domains in emergency medicine. Further, we hope to adapt our strategies to educate a larger, interprofessional audience of nurses, paramedics, law enforcement, and others for future iterations.

## Data Availability

Data sharing is not applicable to this article as no datasets were generated or analyzed during the current study.

## Abbreviations

ABEM: American Board of Emergency Medicine
ACGME: Accreditation Council for Graduate Medical Education
EMS: Emergency medical services
MCI: Mass casualty incident
MCQ: Multiple choice questions
START: Simple Triage and Rapid Treatment
